# Decreased Expression of circ_0000160 in Breast Cancer With Axillary Lymph Node Metastasis

**DOI:** 10.3389/fmolb.2021.690826

**Published:** 2022-02-08

**Authors:** Ya-Wen Wang, Xu Chen, Yaru Tian, Long Liu, Peng Su

**Affiliations:** ^1^ Department of Breast Surgery, General Surgery, Qilu Hospital, Cheeloo College of Medicine, Shandong University, Jinan, China; ^2^ Department of Pathology, Qilu Hospital, Cheeloo College of Medicine, Shandong University, Jinan, China; ^3^ Department of Radiation Oncology, Shandong Cancer Hospital and Institute, Shandong First Medical University and Shandong Academy of Medical Sciences, Jinan, China

**Keywords:** breast cancer, circRNA, lymph node metastasis, circ_0000160, transcriptome sequencing

## Abstract

**Background:** Circular RNAs (circRNAs) have been shown to play important roles in the development and progression of human cancers. Emerging evidence shows that circRNAs have the potential to be promising biomarkers for cancer diagnosis and prognosis. However, the roles of circRNAs in breast cancer axillary lymph node metastasis (ALNM) remain to be determined.

**Methods:** Transcriptome sequencing was utilized to screen the differentially expressed circRNAs in three breast cancer tissues with ALNM and three without ALNM. Differentially expressed circRNAs were further verified by quantitative real-time PCR. Moreover, receiver operating characteristic (ROC) curve analysis was performed to calculate the value of circRNAs to distinguish breast cancer tissues with ALNM and those without ALNM. To explore the potential mechanism of the circRNAs, a circRNA–miRNA–mRNA network was constructed based on the CircInteractome, circBank, and mirDIP online software.

**Results:** In total, 31 differentially expressed circRNAs were identified by transcriptome sequencing; among them, 21 were upregulated and 10 were downregulated in breast cancer with ALNM compared to those without ALNM. Circ_0000160 was validated to be downregulated in breast cancer tissues with ALNM compared with those without ALNM. The ROC curve showed the ability of circ_0000160 to distinguish breast cancer tissues with ALNM and those without ALNM, with an area under the curve of 0.7435. Furthermore, bioinformatics analysis revealed that the predicted mRNAs for circ_0000160 may be related to lymph node metastasis. The predicted mRNAs for circ_0000160 may be involved in many cancer-related pathways.

**Conclusion:** A decreased expression of circ_0000160 was found in breast cancer with axillary lymph node metastasis. Circ_0000160 may have the potential to distinguish breast cancer with axillary lymph node metastasis from those without axillary lymph node metastasis.

## Introduction

Breast cancer is one of the most common malignant tumors and the leading cause of cancer-related death in women. For breast cancer, deaths are usually caused not by the primary tumor but by metastases (Wang et al., 2020; Kim, 2021). Axillary lymph node metastasis (ALNM) is one of the most important prognostic indicators and a crucial component in the staging system (Sawaki et al., 2019) Metastasis to lymph nodes is a high-risk factor for relapse and poor survival (Si et al., 2019). Additionally, identifying metastasis is essential for treatment regimens (Choi et al., 2017). Currently, ideal biomarkers are needed for ALNM prediction (Lee et al., 2018; Chang et al., 2020).

Circular RNAs (circRNAs) are single-stranded, covalently closed RNA molecules and are the downstream products of precursor mRNA back-splicing (Vo et al., 2019). Their high abundance, stability, evolutionary conservation among species, and high cell and tissue specificity endow circRNAs with the potential as molecular biomarkers for cancer diagnosis (Wang S. et al., 2021; Wei et al., 2021). For example, circEHD2, circENGLN3, and circNETO2 were found to be upregulated in clear cell renal carcinoma and showed diagnostic and prognostic values (Frey et al., 2021). Recently, Mani et al. identified hsa_circ_0006743 and hsa_circ_0002496 to be significantly upregulated in early-stage breast cancer tissues compared to normal samples (Rao et al., 2021). We have previously found that circ_0000745, circ_0001531, and circ_0001640 were upregulated in breast cancer, compared with benign tumor and healthy control (Wang Y.-W. et al., 2021). However, the profile of ALNM-related circRNAs in breast cancer has not been well established.

In this study, we investigated the profile of circRNAs using transcriptome sequencing and quantitative real-time PCR (qRT–PCR) in breast cancer tissues with and without ALNM.

## Materials and Methods

### Tissue Samples

For transcriptome sequencing, breast cancer tissues were collected from six patients, including three with ALNM and three without ALNM, at the Qilu Hospital of Shandong University (Shandong, China). The inclusion criteria were breast cancer patients who had undergone mastectomy, were pathologically diagnosed with ALNM or without ALNM, and signed informed consent. Patients who had preoperative chemotherapy or radiotherapy were excluded. Patients with ALNM were matched with patients without ALNM with respect to the timing of the sampling and duration of specimen storage. In the validation stage, another 56 breast cancer tissues (24 with ALNM and 32 without ALNM) were collected from the Qilu Hospital of Shandong University. Typical figures of hematoxylin and eosin (H&E)-stained sections of breast cancer with ALNM and without ALNM are provided in Supplementary Figure S1. All cases of breast cancer were evaluated histologically according to the WHO Classification of Tumors of the Breast (4th edition, 2012) and staged using the Tumor–Node–Metastasis (TNM) staging of the American Joint Committee on Cancer (AJCC, 7th edition, 2010). All fresh specimens were frozen in liquid nitrogen until further use. Written informed consent was obtained from all of the enrolled participants. The present study was approved by the Ethics Committee of the Qilu Hospital of Shandong University.

### Transcriptome Sequencing for circRNA Profiling

Total RNA was isolated from breast cancer tissues using TRIzol reagent (Invitrogen, Carlsbad, CA, United States) according to the manufacturer’s protocol. RNA quality and quantity were measured on a Nanodrop spectrophotometer (ND-1000, Nanodrop Technologies), and RNA integrity was determined by gel electrophoresis. Total RNA was used to remove the rRNAs using Ribo-Zero rRNA Removal Kits (Illumina, United States) following the manufacturer’s instructions. RNA libraries were constructed by using rRNA-depleted RNAs with the TruSeq Stranded Total RNA Library Prep Kit (Illumina, United States) according to the manufacturer’s instructions. Libraries were controlled for quality and quantified using the BioAnalyzer 2,100 system (Agilent Technologies, United States). Ten pM libraries were denatured as single-stranded DNA molecules, captured on Illumina flow cells, amplified *in situ* as clusters, and finally sequenced for 150 cycles on an Illumina HiSeq Sequencer according to the manufacturer’s instructions. Paired-end reads were harvested from an Illumina HiSeq Sequencer after quality filtering. The reads were aligned to the reference genome/transcriptome with Bowtie2 software, and circRNAs were detected and identified with find_circ software. Raw junction reads for all samples were normalized by a total read number and log2 transformed. Differentially expressed circRNAs were identified by the *t* test between two groups. The transcriptome sequencing data were deposited in the Gene Expression Omnibus (GEO) database under the accession number GSE173766.

### Quantitative Real-Time PCR

Briefly, total RNA was extracted from breast cancer tissues using TRIzol reagent (Invitrogen, Carlsbad, CA, United States) according to the manufacturer’s protocol. Reverse transcription was performed using the Invitrogen SuperScript III Reverse Transcriptase kit (Invitrogen). qRT–PCR was performed on a ViiA 7 Real-time PCR system (Applied Biosystems) using the AceQ qPCR SYBR Green Master Mix (Vazyme, Nanjing, China). ACTB levels were used to normalize the expression of circRNAs. The data were analyzed by the 2^−ΔΔCT^ method. Related primers are shown in Supplementary Table S1.

### CircRNA–miRNA–mRNA ceRNA (Competing Endogenous RNA) Network Analysis

The circRNA–miRNA interaction was predicted using CircInteractome (Dudekula et al., 2016) and circBank (Liu et al., 2019). miRNAs shared by the two software programs were selected for further mRNA predictions. The mRNAs targeted by miRNAs were predicted using mirDIP v4.1 (Tokar et al., 2018). The top 10 mRNA targets of miRNAs were first screened based on the integrated score provided by mirDIP. The circRNA–miRNA–mRNA ceRNA network was visualized by Cytoscape software (Shannon et al., 2003). KEGG pathway analysis for mRNAs was performed by the WebGestalt online tool (Liao et al., 2019), and the top 10 pathways based on the *p* value are presented.

### Bioinformatics Analysis

For analysis of the predicted miRNAs, mRNAs and host gene for circ_0000160, miRNA, and mRNA expression profiles and the corresponding clinicopathological information [lymph node metastasis (LNM) status] of the breast cancer patients were obtained from The Cancer Genome Atlas (TCGA) (https://portal.gdc.cancer.gov/). For further analysis, the RNA-sequencing data were normalized as TPM (transcripts per kilobase of exon model per million mapped reads) for mRNA or reads of exon model per million mapped reads for miRNA. Log2-transformed normalized values were used for identification of differentially expressed genes between two groups (with LNM and without LNM) using the Wilcoxon rank sum test.

### Statistical Analysis

Statistical analyses were performed using GraphPad Prism 5.0 (GraphPad Software, Inc., San Diego, CA, United States) and SPSS 20.0 (SPSS, Chicago, IL, United States). The significance of the differences was determined by a nonparametric test between two groups and one-way analysis of variance between multiple groups. The chi-square test or Fisher’s exact test was used, as appropriate, to analyze the relationship between the circRNA expression and clinicopathological variables. *p* < 0.05 was considered statistically significant.

## Results

### Expression Profiling of circRNAs in Breast Cancer With Axillary Lymph Node Metastasis and Without ALNM

**FIGURE 1:**
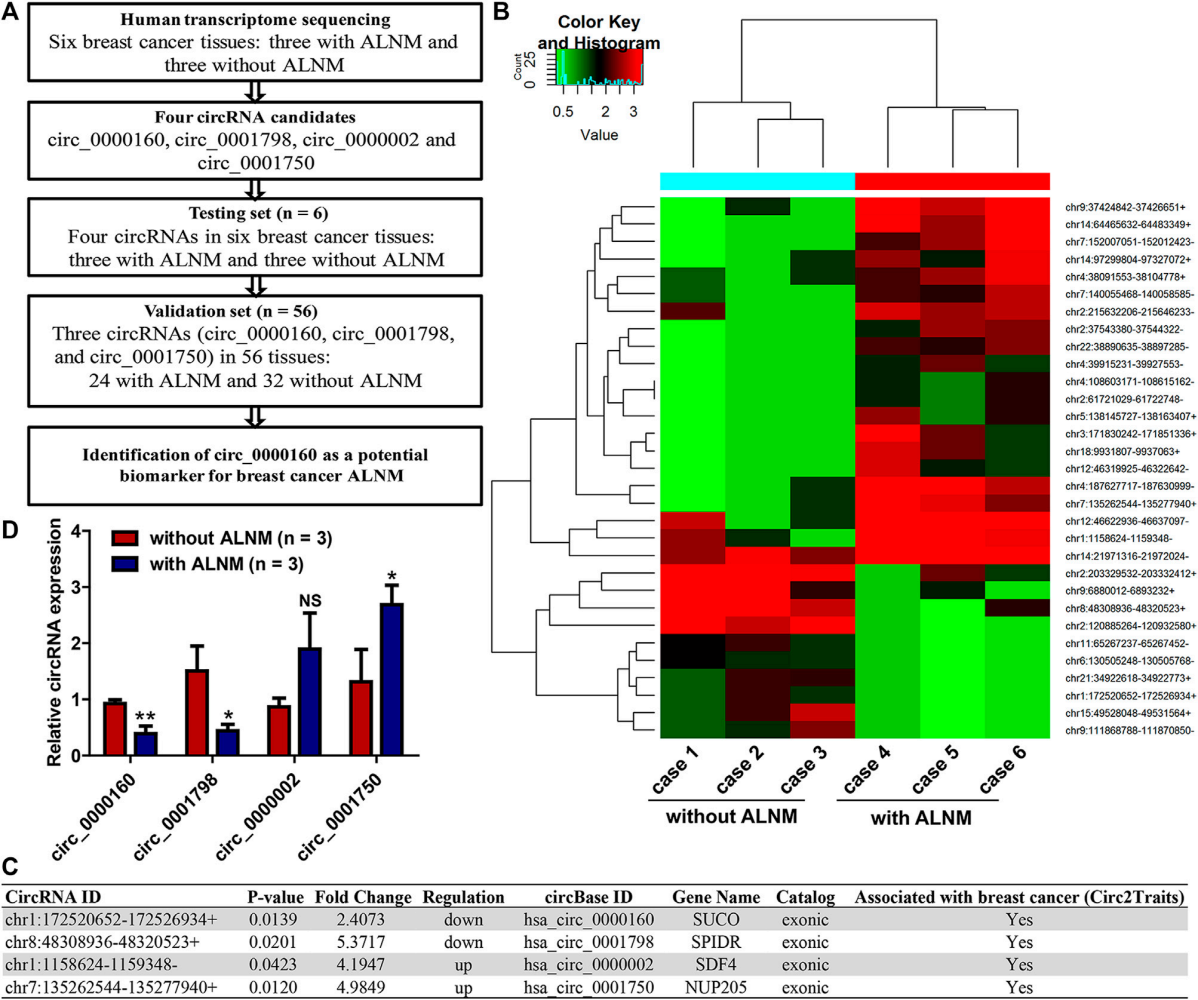
Screening axillary lymph node metastasis (ALNM)-related circRNAs in breast cancer using transcriptome sequencing. **(A)** The flow diagrams of the current study. **(B)** Human transcriptome sequencing was used to screen dysregulated circRNAs associated with breast cancer axillary ALNM in three breast cancer tissues with ALNM and three without ALNM. In total, 21 circRNAs were upregulated and 10 were downregulated in breast cancer with ALNM compared with those without ALNM. **(C)** Four circRNAs were selected for further investigation due to their high raw signal (junction reads) in transcriptome sequencing and potential association with breast cancer determined by the Circ2Traits database. **(D)** qRT–PCR was used to detect the expression of the four circRNAs in three breast cancer tissues with ALNM and three without ALNM. Circ_0000160 and circ_0001798 were downregulated, and circ_0001750 was upregulated in three breast cancer tissues with ALNM compared with three breast cancer tissues without ALNM (*p < 0.05, **p < 0.01). Circ_0000002 did not show significant differences between the two groups.

To identify dysregulated circRNAs associated with breast cancer ALNM, transcriptome sequencing was performed on three breast cancer tissues with ALNM and three without ALNM (Figure 1A). In total, 31 circRNAs were found to be differentially expressed in breast cancer with ALNM vs. without ALNM (fold change >2, *p* < 0.05) (Figure 1B). Among the 31 circRNAs, 21 were upregulated and 10 were downregulated in breast cancer with ALNM compared with those without ALNM.

Next, we utilized Circ2Traits (Ghosal et al., 2013), a database collecting disease-associated circRNAs based on bioinformatics analysis and publications, to screen potential disease-associated circRNAs. Nine circRNAs were included in the Circ2Traits database, which were supposed to be associated with human cancers, cardiac hypertrophy, nonalcoholic fatty liver disease, Duchenne muscular dystrophy, and other human diseases. Finally, four of the nine circRNAs were selected for further investigation. These four circRNAs (upregulated: circ_0000002 and circ_0001750; downregulated: circ_0000160, circ_0001798) showed relatively high raw signals (junction reads) in transcriptome sequencing and were thought to be associated with breast cancer, as determined by the Circ2Traits database (Figure 1C).

### Validation of Differentially Expressed circRNAs Using qRT–PCR

First, qRT–PCR was used to detect the expression of circ_0000002 and circ_0001750, circ_0000160, and circ_0001798 in the testing set (the six patients used in the transcriptome sequencing). The data showed that circ_0000160 and circ_0001798 were downregulated and that circ_0001750 was upregulated in three breast cancer tissues with ALNM compared with three breast cancer tissues without ALNM (Figure 1D, *p* < 0.05). The expression of circ_0000002 did not show significant differences between the two groups (Figure 1D).

**FIGURE 2:**
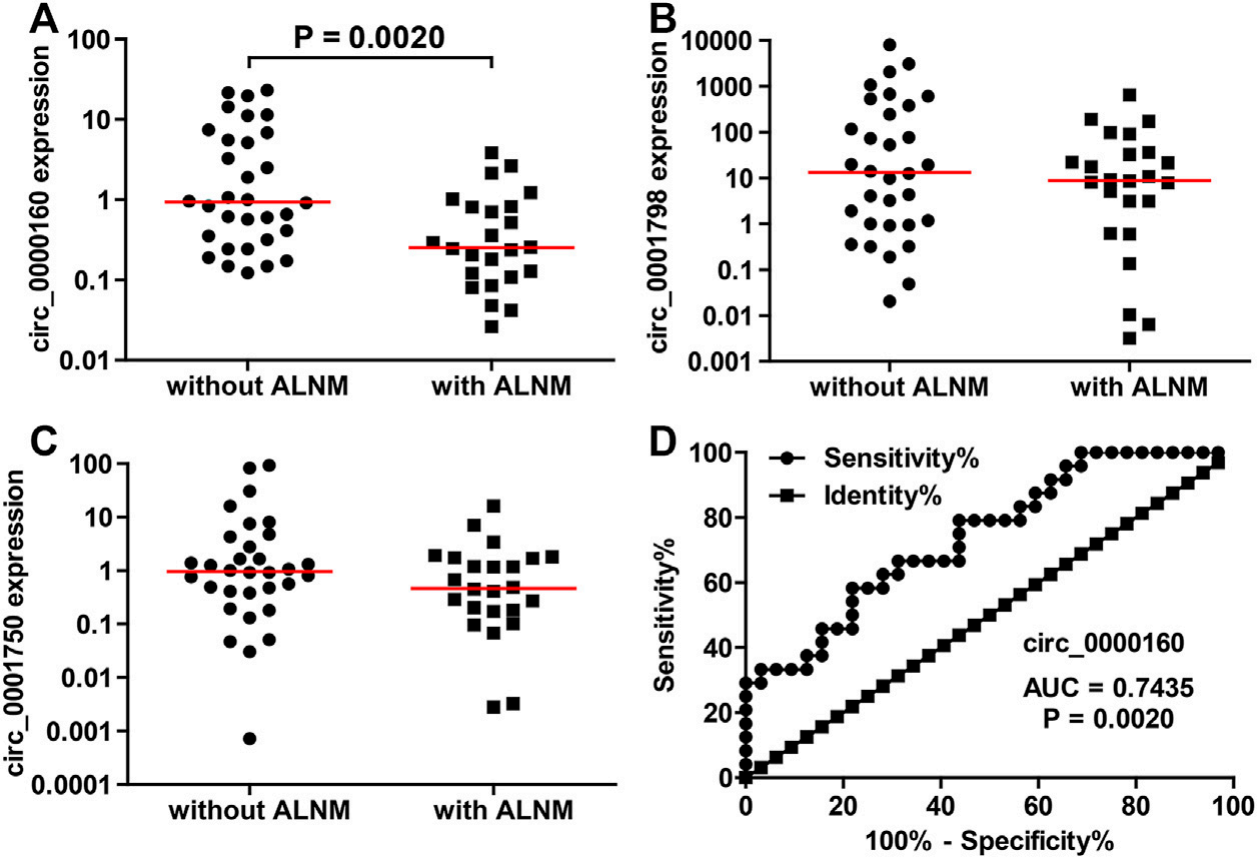
Decreased expression of circ_0000160 was found in breast cancer with ALNM in the validation cohort containing 56 patients. **(A)** Circ_0000160 was downregulated in 24 breast cancer tissues with ALNM compared to 32 tissues without ALNM. **(B,C)** Circ_0001798 and circ_0001750 did not show significant differences between the two groups. **(D)** ROC analysis showed that circ_0000160 had an area under the curve of 0.7435 (*p* = 0.0020) to discriminate tissues with ALNM from those without ALNM.

**FIGURE 3:**
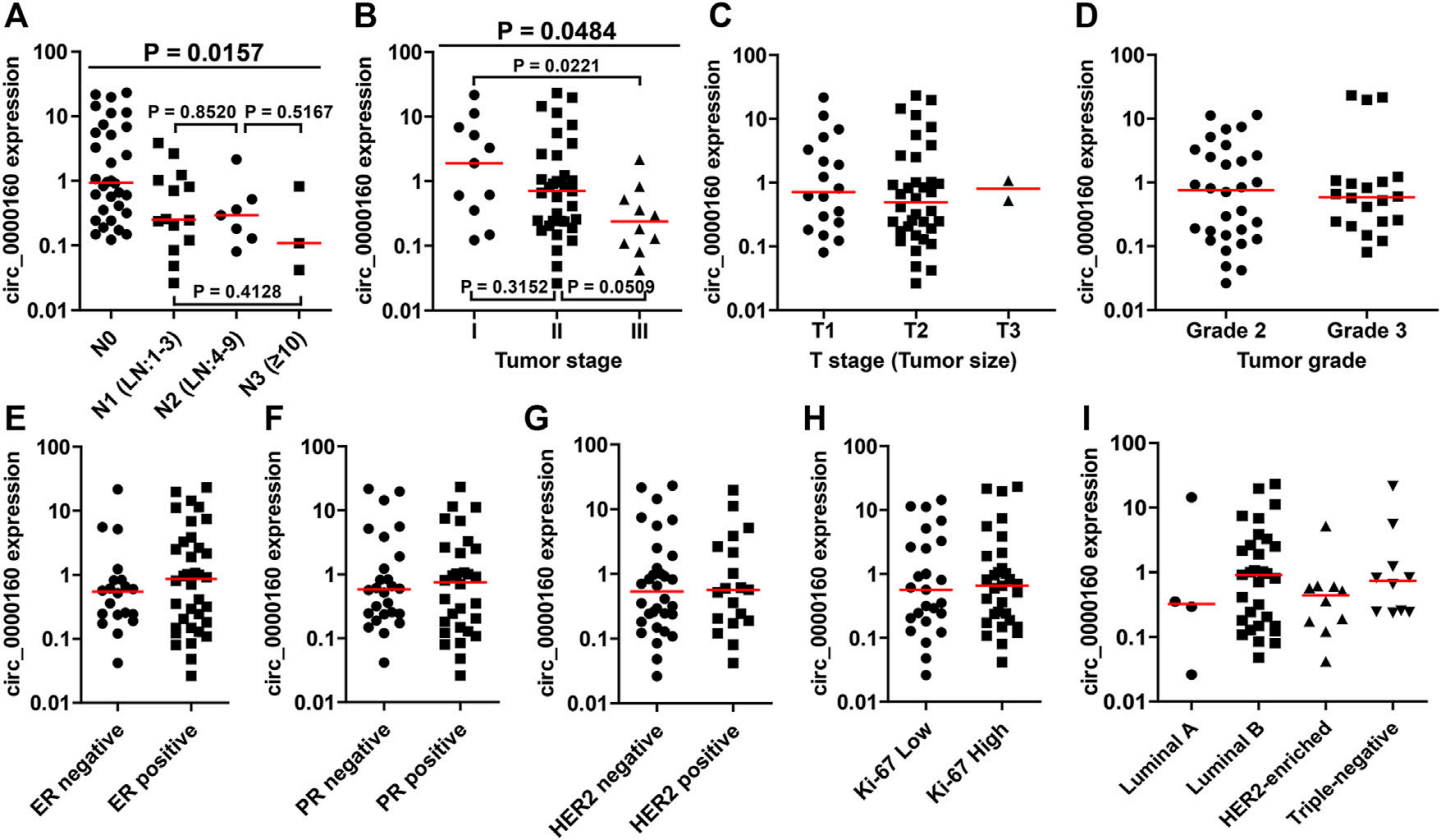
Association between the circ_0000160 expression and clinicopathological parameters in the validation cohort containing 56 patients. **(A)** A decreasing trend of circ_0000160 was found in breast cancer tissues with more advanced N stages (increasing ALNM number, one-way analysis of variance, *p* = 0.0157); however, the comparison for each two stages did not reach statistical significance (N1 vs. N2, *p* = 0.8520; N1 vs. N3, *p* = 0.4128; N2 vs. N3, *p* = 0.5176). **(B)** Circ_0000160 was even decreased in breast cancer with a more advanced tumor stage (one-way analysis of variance, *p* = 0.0484; stage I vs. stage II, *p* = 0.3152; II vs. III, *p* = 0.0509; I vs. III, *p* = 0.0221). **(C)-(I)** Circ_0000160 expression showed no relationship with the tumor size, tumor grade, ER/PR/HER2/Ki-67 expression, or molecular type (ER, estrogen receptor; PR, progesterone receptor).

Then, the differential expression of circ_0000160, circ_0001798, and circ_0001750 was further validated in a larger cohort containing 56 patients. Only circ_0000160 was downregulated in 24 breast cancer tissues with ALNM compared with 32 breast cancer tissues without ALNM (Figures 2A–C). The receiver operating characteristic (ROC) curve reflected strong separation between tissues with ALNM and those without ALNM, with an area under the curve (AUC) of 0.7435 (*p* = 0.0020) (Figure 2D). A decreasing trend of circ_0000160 was found in breast cancer tissues with more advanced N stages (increasing ALNM number, Figure 3A, one-way analysis of variance, *p* = 0.0157); however, the comparison for every two stages did not reach statistical significance (N1 vs. N2, *p* = 0.8520; N1 vs. N3, *p* = 0.4128; N2 vs. N3, *p* = 0.5176).

### Association Between the circRNA Expression and Clinicopathological Parameters of Breast Cancer Patients

To further determine the clinical significance of circRNAs, we analyzed the association between the circRNA expression and the clinicopathological parameters of breast cancer patients (Table 1). Here, the patients were divided into a high-expression group and a low-expression group based on the median expression value of circRNA. Our results showed that the low expression of circ_0000160 was correlated with axillary lymph node metastasis (*p* = 0.031). Additionally, circ_0000160 was even decreased in breast cancer with a more advanced tumor stage (Figure 3B, one-way analysis of variance, *p* = 0.0484; Stage I vs. Stage II, *p* = 0.3152; II vs. III, *p* = 0.0509; I vs. III, *p* = 0.0221). For circ_0001750, a higher expression was found in breast cancer tissues with a higher tumor grade, negative progesterone receptor (PR), or higher Ki-67 expression (Supplementary Figure S2; Table 1). No association was found between the circ_0001798 expression and any clinicopathological parameters (Supplementary Figure S3).

**TABLE 1:** Association between the circRNA expression and clinicopathologic features in breast cancer.

Variables	circ_0000160		*p*	circ_0001798		*p*	circ_0001750		*p*
	Low	High		Low	High		Low	High	
Age (y)									
≤50	12	14		12	14		12	14	
>50	16	11	0.592	16	14	0.592	16	14	0.592
Tumor grade									
II	14	16		16	14		18	12	
III	11	9	0.564	10	10	0.817	9	11	0.297
Unknown	6			6			6		
Lymph node metastasis									
Negative	12	20		15	17		14	18	
Positive	16	8	0.031	13	11	0.589	14	10	0.280
ER									
Negative	12	8		12	8		8	12	
Positive	16	20	0.265	16	20	0.265	20	16	0.265
PR									
Negative	14	12		14	12		9	17	
Positive	14	16	0.592	14	16	0.592	19	11	0.032
HER2									
Negative	16	16		17	15		16	16	
Positive	11	8	0.585	8	11	0.447	8	11	0.585
Unknown	5			5			5		
Ki-67									
≤30%	13	12		13	12		16	9	
>30%	15	16	0.788	15	16	0.788	12	19	0.060
p53									
Negative	15	14		14	15		16	13	
Positive	12	14	0.680	13	13	0.898	11	15	0.341
Unknown	1			1			1		
T stage									
1	8	10		6	12		6	12	
2	19	17		21	15		21	15	
3	1	1	0.846	1	1	0.223	1	1	0.223
N stage									
0	12	20		15	17		14	18	
1	8	6		8	6		8	6	
2	6	1		3	4		4	3	
3	2	1	0.103	2	1	0.829	2	1	0.738
Tumor stage									
I	4	7		3	8		3	8	
II	16	19		20	15		19	16	
III	8	2	0.097	5	5	0.225	6	4	0.231
Molecular subtypes									
Luminal A	3	1		1	3		2	2	
Luminal B	13	18		15	16		17	14	
HER2-enriched	8	2		6	4		4	6	
Triple-negative	4	6	0.122	6	4	0.612	4	6	0.784
Unknown	1			1			1		

### CircRNA–miRNA–mRNA ceRNA Network Construction

**FIGURE 4:**
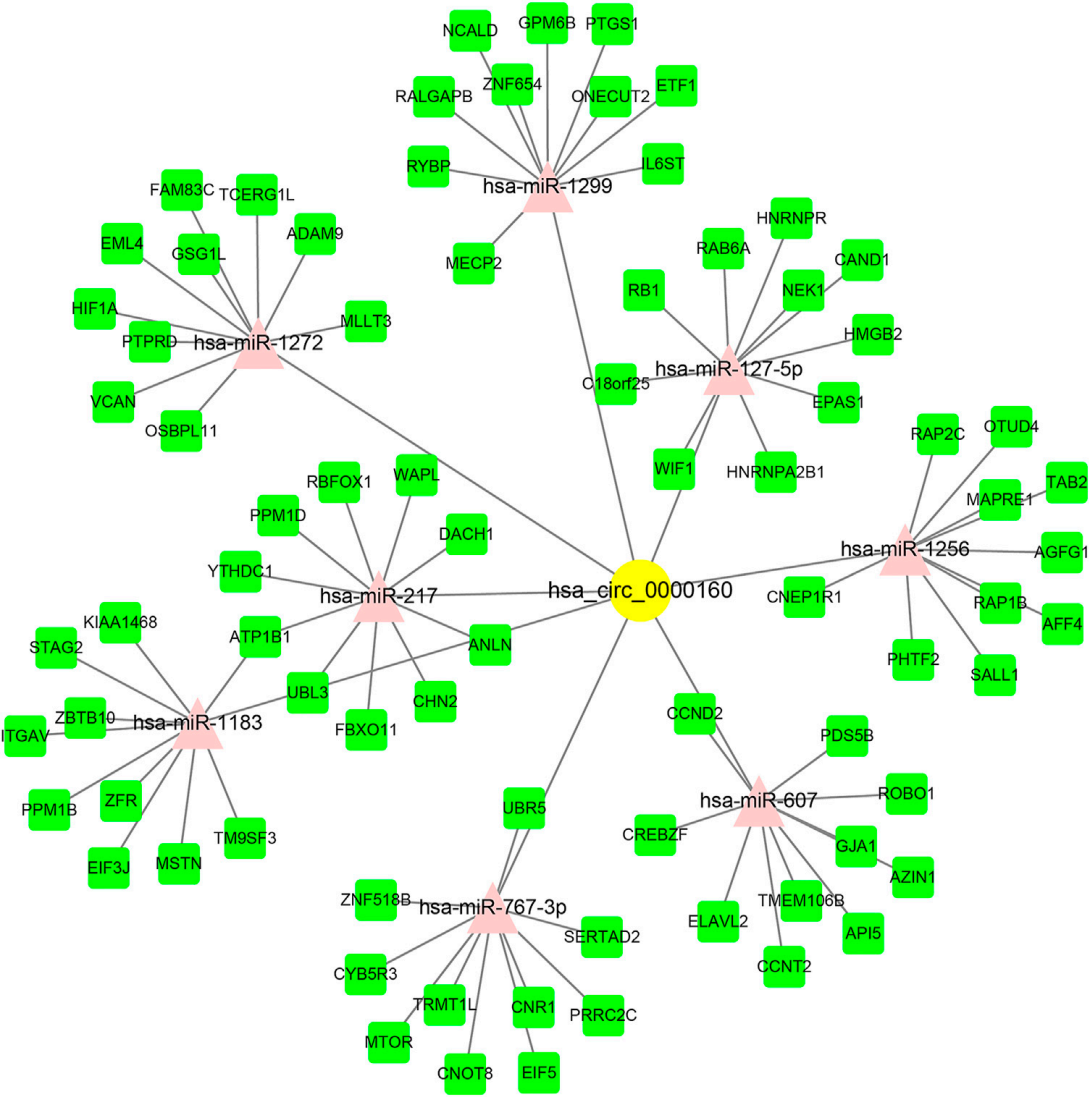
circRNA–miRNA–mRNA ceRNA network for circ_0000160. miRNAs were predicted by CircInteractome and circBank. mRNAs were predicted using mirDIP v4.1. The top 10 mRNA targets of each miRNA were selected based on the integrated score provided by mirDIP. Cytoscape software was used to visualize the network.

Circ_0000160 was subjected to further bioinformatics analysis to explore the potential mechanisms in breast cancer. Based on the prediction of CircInteractome and circBank, eight miRNAs (miR-607, miR-127-5p, miR-1272, miR-1299, miR-1183, miR-217, miR-767-3p, and miR-1256) could potentially interact with circ_0000160. Then, the mRNA targets of these miRNAs were predicted using mirDIP v4.1, and the top 10 mRNAs of each miRNA were included in the circRNA–miRNA–mRNA network construction (Figure 4). The potential targets of miRNAs were enriched in cancer-related pathways, such as pathways in cancer, the cell cycle, cellular senescence, and the JAK-STAT signaling pathway (Table 2).

**TABLE 2:** KEGG analysis of circ_0000160 based on the ceRNA network.

Description	Enrichment ratio	*p* value
Pathways in cancer	3.47	0.0015
Thyroid hormone signaling pathway	7.84	0.0016
Renal cell carcinoma	9.88	0.0033
Kaposi sarcoma-associated herpesvirus infection	4.89	0.0085
Th17 cell differentiation	6.37	0.0113
Cell cycle	5.50	0.0168
Cellular senescence	4.26	0.0325
Central carbon metabolism in cancer	6.99	0.0329
JAK-STAT signaling pathway	4.21	0.0336
Non–small cell lung cancer	6.89	0.0338

**FIGURE 5:**
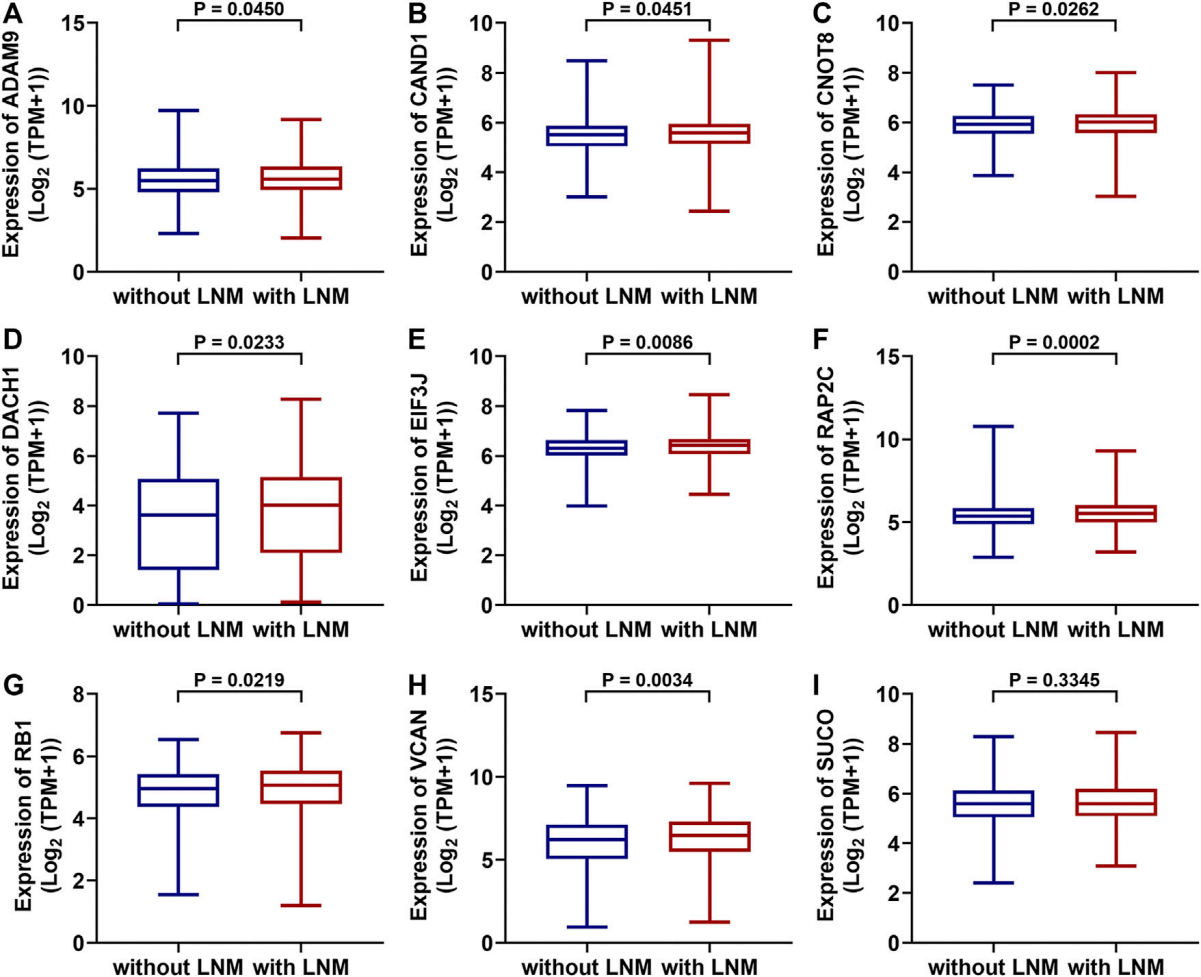
Differentially expressed mRNAs between breast cancer with lymph node metastasis and those without lymph node metastasis based on the TGCA dataset (BRCA). ADAM9, CAND1, CNOT8, DACH1, EIF3J, RAP2C, RB1, and VCAN were upregulated in breast cancer with lymph node metastasis (*n* = 551) compared with those without lymph node metastasis (**A**-**H**, *n* = 505). The SUCO expression showed no obvious difference in breast cancer with lymph node metastasis and those without lymph node metastasis (**I**).

TCGA data were used to test whether the predicted miRNAs and mRNAs (and the host gene SUCO) for circ_0000160 are deregulated in breast cancers with lymph node metastasis. Among the predicted miRNAs and mRNAs, ADAM9, CAND1, CNOT8, DACH1, EIF3J, RAP2C, RB1, and VCAN were found to be deregulated in breast cancer with lymph node metastasis compared with those without lymph node metastasis (Figures 5A-H). Of note, DACH1 and VCAN showed obvious upregulation in breast cancer with lymph node metastasis. However, the host gene SUCO (Figure 5I) and the analyzed miRNAs (data not shown) showed no obvious difference in breast cancer with lymph node metastasis and those without lymph node metastasis.

## Discussion

Breast cancer is one of the most common cancers. Recently, circRNAs have shown emerging promise as potential disease biomarkers (Verduci et al., 2021). Based on transcriptome sequencing followed by qRT–PCR confirmation, we identified circ_0000160 as significantly associated with ALNM in breast cancer. Circ_0000160 showed a relatively high predictive value to distinguish breast cancer patients with and without ALNM. It is noted that three pairs were evaluated by transcriptome sequencing as a discovery set, which may result in bias, which means that a small number of samples in high-throughput screening may produce “false positive” and “false negative” results. First, potentially differentially expressed circRNAs revealed by transcriptome sequencing may not be truly differential and should be further validated in a larger cohort. Second, we cannot exclude the possibility that there are other ALNM-related circRNAs other than the 31 circRNAs revealed by our transcriptome sequencing data. More ALNM-related circRNAs should be explored with a larger cohort using high-throughput technology.

Circ_0000160 is a novel circRNA that has not ever been investigated in human diseases, including cancers. The host gene SUCO (the SUN domain containing the ossification factor) has rarely been investigated in human cancers. Harland et al. found that mice lacking SUCO showed impaired bone formation and spontaneous fractures, and their results suggested SUCO as a candidate gene for brittle bone disorders (Sohaskey et al., 2010). Recently, exome sequencing identified SUCO mutations in mesial temporal lobe epilepsy, and a lack of SUCO led to abnormal development of neurons (Sha et al., 2015). SUCO was found to be upregulated in hepatocellular carcinoma (Yue et al., 2019) and may be targeted by miR-497 (Chen et al., 2021). Our data showed that circ_0000160 was downregulated, while the host gene SUCO showed no significant change in breast cancer with lymph node metastasis compared with those without lymph node metastasis. Recently, Kristensen et al. found that the expression of circRNAs does not often correlate well with the expression of host genes linearly (Kristensen et al., 2018). This argues that circRNAs are not merely steady-state byproducts of mRNA splicing but rather the product of a new type of regulated alternative splicing. We then analyzed the isoform expression data of the SUCO gene in breast cancer based on the GEPIA2 database (Tang et al., 2017). SUCO has 10 main isoforms (transcripts), and they showed distinct expression distributions in breast cancer (Supplementary Figure S4A). Overall, SUCO was upregulated in breast cancer tissues compared with normal breast tissues (Supplementary Figure S4B). However, some transcripts (e.g., ENST00000263688.3, circ_0000160 was derived from this transcript) were upregulated, some (e.g., ENST00000608566.1) showed no obvious change, and some (e.g., ENST00000609685.1) even showed a downregulation trend in breast cancer (Supplementary Figures S4C–L). These results suggested that different isoforms may show distinct expression patterns. Further investigation on the function and mechanism of circ_0000160, as well as its relationship with SUCO, is needed in the future.

There were a few limitations in our study. First, the sample size was small. Circ_0000160 and the other two circRNAs circ_0001798 and circ_0001750, which did not show a relationship with ALNM, need further confirmation, ideally, with varied geographic and ethnic populations, with different stages, subtypes, and treatment regimens. Second, the present study mainly focused on clinically significant circRNAs in breast cancer. The function and mechanism of circRNAs in breast cancer need further investigation. Third, a single marker is not sufficiently accurate to predict ALNM. In future studies, the integration of multiple biomarkers to predict lymph node metastasis may supersede their individual predictive value.

In conclusion, we revealed the circRNA profile related to breast cancer ALNM using transcriptome sequencing. A decreased expression of circ_0000160 was found in breast cancer with axillary lymph node metastasis. Bioinformatics analysis showed that circ_0000160 may interact with numerous miRNAs and mRNAs and participate in many cancer-related pathways. The predicted mRNAs for circ_0000160 may be related to lymph node metastasis. This study provides a framework for understanding the mechanisms of breast cancer ALNM from the perspective of circRNAs. Circ_0000160 may have the potential to distinguish breast cancer with axillary lymph node metastasis from those without axillary lymph node metastasis.

## Data Availability

The datasets presented in this study can be found in online repositories. The names of the repository/repositories and accession number(s) can be found below: https://www.ncbi.nlm.nih.gov/geo/query/acc.cgi?acc=GSE173766.

## Supplementary Material

Supplementary Figure S1 Typical figures of hematoxylin and eosin (H&E)-stained sections of breast cancer with ALNM (axillary lymph node metastasis) and without ALNM. Two cases were used as examples, and their sections of primary breast cancer and the corresponding axillary lymph node were provided **(A,B,E,F**, primary breast invasive ductal carcinoma; **C,D**, without ALNM; **G,H,** with ALNM) **(A,C,E,G**, magnification × 40; **B,D,F,H**, magnification × 200**)**.

Supplementary Figure S2 Association between the circ_0001750 expression and clinicopathological parameters in the validation cohort containing 56 patients. Circ_0001750 displayed a higher expression in tissues with a higher grade (grade 3 vs. grade 2), negative PR status, or higher Ki-67 expression. The circ_0001750 expression showed no relationship with other parameters (ER, estrogen receptor; PR, progesterone receptor).

Supplementary Figure S3 Circ_0001798 expression showed no relationship with clinicopathological parameters in the validation cohort containing 56 patients (ER, estrogen receptor; PR, progesterone receptor).

Supplementary Figure S4 Isoform expression data of the SUCO gene in breast cancer based on the GEPIA2 database. **(A)** Isoform expression distribution of SUCO in breast cancer. **(B–L)** Comparison of the SUCO isoform expression in breast cancer and normal tissues (**p* < 0.01).

## References

[B1] Chang J. M. Leung J. W. T. Moy L. Ha S. M. Moon W. K. (2020). Axillary Nodal Evaluation in Breast Cancer: State of the Art. Radiology 295 (3), 500–515. 10.1148/radiol.2020192534 32315268

[B2] Chen S. Fu Z. Wen S. Yang X. Yu C. Zhou W. (2021). Expression and Diagnostic Value of miR-497 and miR-1246 in Hepatocellular Carcinoma. Front. Genet. 12, 666306. 10.3389/fgene.2021.666306 34163524 PMC8215616

[B3] Choi H. Y. Park M. Seo M. Song E. Shin S. Y. Sohn Y.-M. (2017). Preoperative Axillary Lymph Node Evaluation in Breast Cancer. Ultrasound Q. 33 (1), 6–14. 10.1097/ruq.0000000000000277 28187012

[B4] Dudekula D. B. Panda A. C. Grammatikakis I. De S. Abdelmohsen K. Gorospe M. (2016). CircInteractome: A Web Tool for Exploring Circular RNAs and Their Interacting Proteins and microRNAs. RNA Biol. 13 (1), 34–42. 10.1080/15476286.2015.1128065 26669964 PMC4829301

[B5] Frey L. Klümper N. Schmidt D. Kristiansen G. Toma M. Ritter M. (2021). CircEHD2, CircNETO2 and CircEGLN3 as Diagnostic and Prognostic Biomarkers for Patients with Renal Cell Carcinoma. Cancers 13 (9), 2177. 10.3390/cancers13092177 33946584 PMC8124893

[B6] Ghosal S. Das S. Sen R. Basak P. Chakrabarti J. (2013). Circ2Traits: a Comprehensive Database for Circular RNA Potentially Associated with Disease and Traits. Front. Genet. 4, 283. 10.3389/fgene.2013.00283 24339831 PMC3857533

[B7] Kim M. Y. (2021). Breast Cancer Metastasis. Adv. Exp. Med. Biol. 1187, 183–204. 10.1007/978-981-32-9620-6_9 33983579

[B8] Kristensen L. S. Hansen T. B. Venø M. T. Kjems J. (2018). Circular RNAs in Cancer: Opportunities and Challenges in the Field. Oncogene 37 (5), 555–565. 10.1038/onc.2017.361 28991235 PMC5799710

[B9] Lee J.-H. Jeong H. Choi J.-W. Oh H. E. Kim Y.-S. (2018). Liquid Biopsy Prediction of Axillary Lymph Node Metastasis, Cancer Recurrence, and Patient Survival in Breast Cancer. Medicine (Baltimore) 97 (42), e12862. 10.1097/md.0000000000012862 30334995 PMC6211877

[B10] Liao Y. Wang J. Jaehnig E. J. Shi Z. Zhang B. (2019). WebGestalt 2019: Gene Set Analysis Toolkit with Revamped UIs and APIs. Nucleic Acids Res. 47 (W1), W199–w205. 10.1093/nar/gkz401 31114916 PMC6602449

[B11] Liu M. Wang Q. Shen J. Yang B. B. Ding X. (2019). Circbank: a Comprehensive Database for circRNA with Standard Nomenclature. RNA Biol. 16 (7), 899–905. 10.1080/15476286.2019.1600395 31023147 PMC6546381

[B12] Rao A. K. D. M. Arvinden V. R. Ramasamy D. Patel K. Meenakumari B. Ramanathan P. (2021). Identification of Novel Dysregulated Circular RNAs in Early-Stage Breast Cancer. J. Cel. Mol. Med. 25 (8), 3912–3921. 10.1111/jcmm.16324 PMC8051735 33544410

[B13] Sawaki M. Shien T. Iwata H. (2019). TNM Classification of Malignant Tumors (Breast Cancer Study Group). Jpn. J. Clin. Oncol. 49 (3), 228–231. 10.1093/jjco/hyy182 30541035

[B14] Sha Z. Sha L. Li W. Dou W. Shen Y. Wu L. (2015). Exome Sequencing Identifies SUCO Mutations in Mesial Temporal Lobe Epilepsy. Neurosci. Lett. 591, 149–154. 10.1016/j.neulet.2015.02.009 25668491

[B15] Shannon P. Markiel A. Ozier O. Baliga N. S. Wang J. T. Ramage D. (2003). Cytoscape: a Software Environment for Integrated Models of Biomolecular Interaction Networks. Genome Res. 13 (11), 2498–2504. 10.1101/gr.1239303 14597658 PMC403769

[B16] Si P. Zhang P. Chen T. Liu G. Lu H. Chen H. (2019). Positive Nonsentinel Lymph Nodes Are Associated with Poor Survival in Breast Cancer: Results from a Retrospective Study. Clin. Transl. Oncol. 21 (8), 1085–1092. 10.1007/s12094-018-02031-5 30632009

[B17] Sohaskey M. L. Jiang Y. Zhao J. J. Mohr A. Roemer F. Harland R. M. (2010). Osteopotentia Regulates Osteoblast Maturation, Bone Formation, and Skeletal Integrity in Mice. J. Cel. Biol. 189 (3), 511–525. 10.1083/jcb.201003006 PMC2867309 20440000

[B18] Tang Z. Li C. Kang B. Gao G. Li C. Zhang Z. (2017). GEPIA: a Web Server for Cancer and normal Gene Expression Profiling and Interactive Analyses. Nucleic Acids Res. 45 (W1), W98–w102. 10.1093/nar/gkx247 28407145 PMC5570223

[B19] Tokar T. Pastrello C. Rossos A. E. M. Abovsky M. Hauschild A.-C. Tsay M. (2018). mirDIP 4.1-integrative Database of Human microRNA Target Predictions. Nucleic Acids Res. 46 (D1), D360–d370. 10.1093/nar/gkx1144 29194489 PMC5753284

[B20] Verduci L. Tarcitano E. Strano S. Yarden Y. Blandino G. (2021). CircRNAs: Role in Human Diseases and Potential Use as Biomarkers. Cell Death Dis. 12 (5), 468. 10.1038/s41419-021-03743-3 33976116 PMC8113373

[B21] Vo J. N. Cieslik M. Zhang Y. Shukla S. Xiao L. Zhang Y. (2019). The Landscape of Circular RNA in Cancer. Cell 176 (4), 869–881. e813. 10.1016/j.cell.2018.12.021 30735636 PMC6601354

[B22] Wang L. Zhang S. Wang X. (2020). The Metabolic Mechanisms of Breast Cancer Metastasis. Front. Oncol. 10, 602416. 10.3389/fonc.2020.602416 33489906 PMC7817624

[B23] Wang S. Zhang K. Tan S. Xin J. Yuan Q. Xu H. (2021a). Circular RNAs in Body Fluids as Cancer Biomarkers: the New Frontier of Liquid Biopsies. Mol. Cancer 20 (1), 13. 10.1186/s12943-020-01298-z 33430880 PMC7798340

[B24] Wang Y.-W. Xu Y. Wang Y.-Y. Zhu J. Gao H.-D. Ma R. (2021b). Elevated circRNAs Circ_0000745, Circ_0001531 and Circ_0001640 in Human Whole Blood: Potential Novel Diagnostic Biomarkers for Breast Cancer. Exp. Mol. Pathol. 121, 104661. 10.1016/j.yexmp.2021.104661 34139239

[B25] Wei G. Zhu J. Hu H.-B. Liu J.-Q. (2021). Circular RNAs: Promising Biomarkers for Cancer Diagnosis and Prognosis. Gene 771, 145365. 10.1016/j.gene.2020.145365 33346098

[B26] Yue C. Liang C. Ge H. Yan L. Xu Y. Li G. (2019). SUCO as a Promising Diagnostic Biomarker of Hepatocellular Carcinoma: Integrated Analysis and Experimental Validation. Med. Sci. Monit. 25, 6292–6303. 10.12659/msm.915262 31434866 PMC6716297

