# Supplementary material for: Decreased Expression of circ_0000160 in Breast Cancer With Axillary Lymph Node Metastasis
Source: Front Mol Biosci. 2022 Feb 8;8:690826. doi: 10.3389/fmolb.2021.690826 (PMC8861307; doi:10.3389/fmolb.2021.690826)
Supplement: Supplementary file 1 [file Table1.docx]

Supplementary Material

| **Supplementary Table S1. circRNA primers used for qRT-PCR analysis.** | | |
| --- | --- | --- |
| **Gene name** | **Primer sequences** | |
| circ_0000160 | Forward | GGTGGTGATCCAAAATCTGC |
|  | Reverse | TGATTCGGCATTGATAGGTC |
| circ_0001798 | Forward | GGCTTTCACACTGAGGTTCC |
|  | Reverse | TGATCGAGAAGGCTTCCTGT |
| circ_0000002 | Forward | TTGGCGAGTAAAGGCCATAG |
|  | Reverse | GCGGAAGTGTGTCTTGCTCT |
| circ_0001750 | Forward | TCGGAAGGATCTTCCAAGTG |
|  | Reverse | CCTAAAGGTGACTGGCAAGC |
| ACTB | Forward | GTGGCCGAGGACTTTGATTG |
|  | Reverse | CCTGTAACAACGCATCTCATATT |
