# Supplementary figures and images for: Decreased Expression of circ_0000160 in Breast Cancer With Axillary Lymph Node Metastasis
Source: Front Mol Biosci. 2022 Feb 8;8:690826. doi: 10.3389/fmolb.2021.690826 (PMC8861307; doi:10.3389/fmolb.2021.690826)

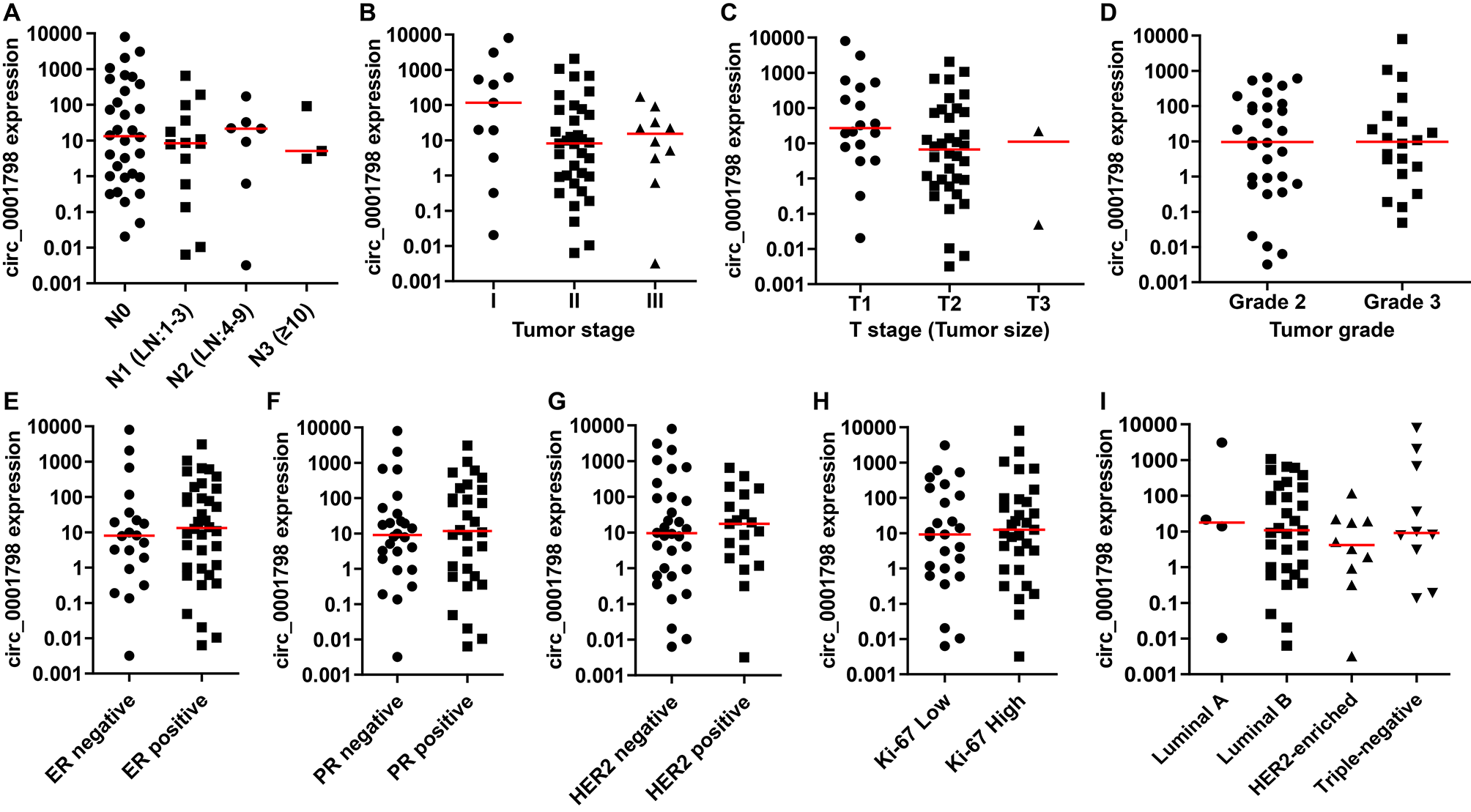

Supplement: Supplementary file 2 [file Image3.TIF]

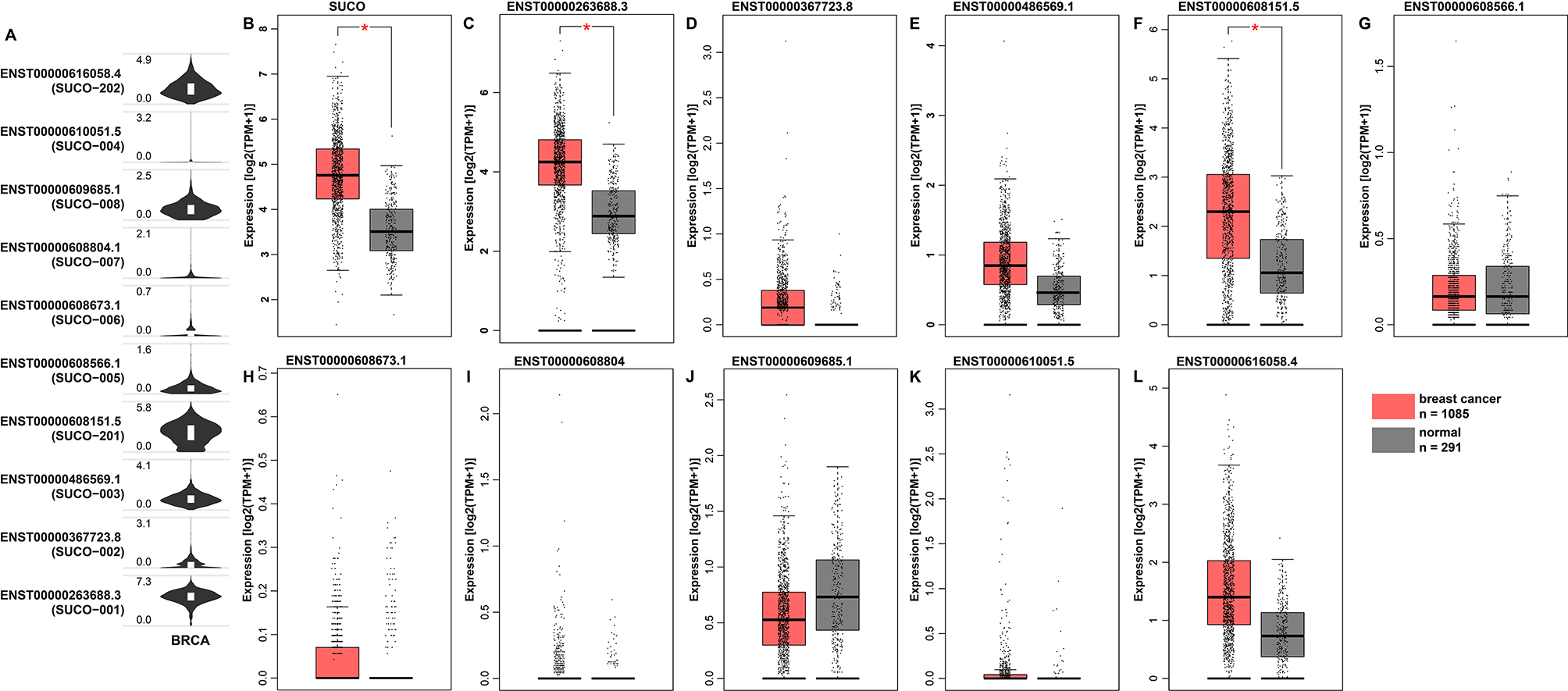

Supplement: Supplementary file 3 [file Image4.TIF]

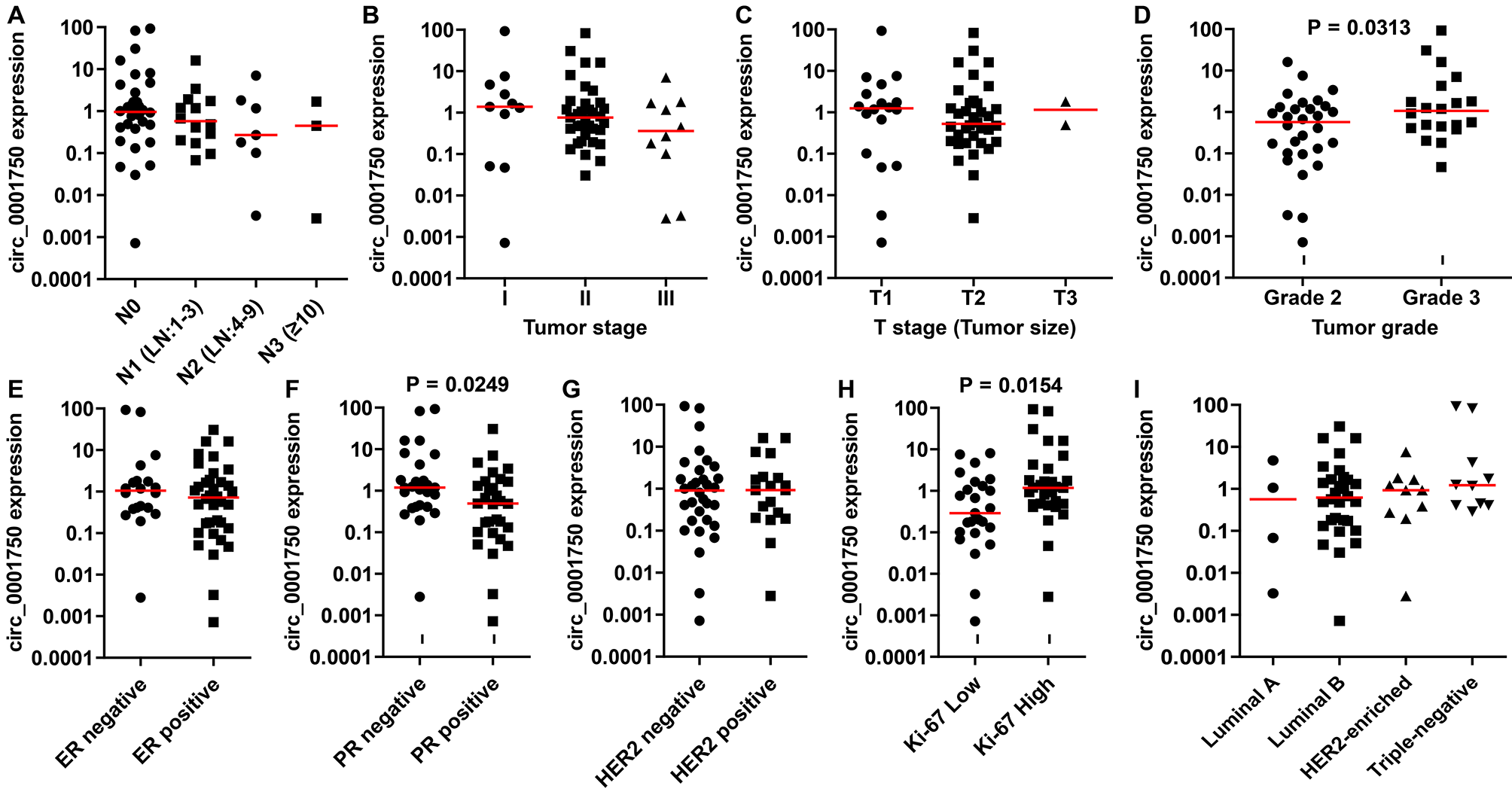

Supplement: Supplementary file 4 [file Image2.TIF]

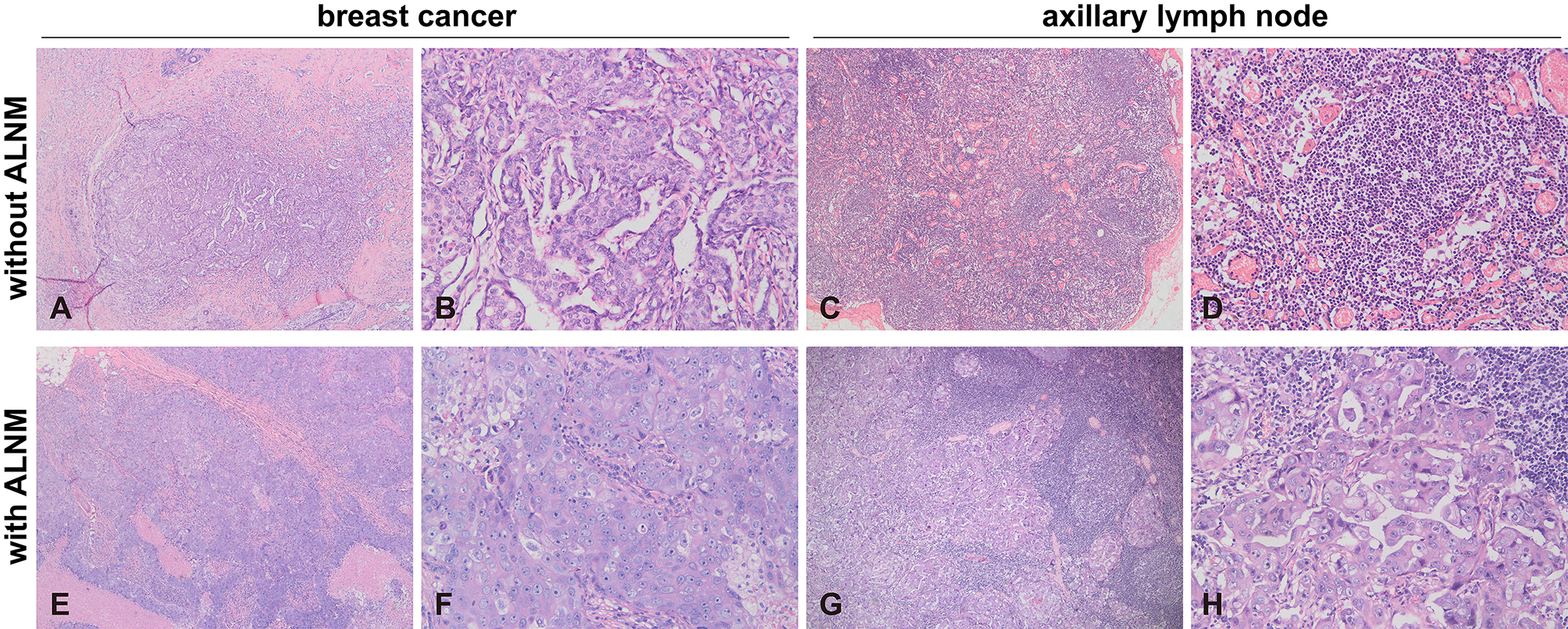

Supplement: Supplementary file 5 [file Image1.TIF]
